# Coping and adaptation strategies among young persons living with type 1 diabetes and their caregivers: textual and photovoice analyses

**DOI:** 10.1186/s12889-023-16573-z

**Published:** 2023-09-01

**Authors:** Bernard Afriyie Owusu, Prince Ofori-Boateng, David Teye Doku

**Affiliations:** 1 Department of Population and Health, University of Cape Coast, Cape Coast, Ghana; 2 Cape Coast Teaching Hospital, Central Region, Cape Coast, Ghana

**Keywords:** Adolescence, Stigma, Social support, Adaptation, Relationship, Youth

## Abstract

**Background:**

Management of type 1 diabetes (T1D) is complex and demanding. It requires patients and their caregivers, particularly those in low-resource settings to adopt productive coping strategies to achieve ideal glycaemic control. Coping and adaptation strategies have far-reaching implications on their behavioural and health outcomes. Yet, it is uncertain how young people living with T1D and their caregivers in low-resource settings cope and adapt to the challenges of T1D management. This study analysed textual and photo evidence on the coping and adaptation strategies employed by young persons living with T1D (warriors) and their caregivers in Ghana.

**Methods:**

Qualitative data were collected from 28 warriors, 12 caregivers, 6 healthcare providers and other stakeholders in southern Ghana using semi-structured interview guides. Participants were identified at T1D support group centres, hospitals, and their places of residence, and recruited into the study using maximum variation and snowball sampling approaches. Data were collected via face-to-face interviews, photovoice, telephone interviews and videoconferencing and were thematically analysed using QSR NVivo 11.

**Results:**

Four superordinate themes which are productive coping, non-productive coping, keeping T1D a secret, and coping with costs of care were identified. Productive coping entailed condition acceptance, planning ahead, seeking social support, borrowing insulin, and overcoming the barriers of insulin storage. On the other hand, avoidance, disengagement, and re-use of syringes were the common non-productive coping approaches. Due to stigma and discrimination, the warriors shrouded their condition in secrecy. As a response to the financial burden of T1D care, caregivers/patients borrowed money, took loans, and sold household items.

**Conclusion:**

Young persons living with T1D and their caregivers adopted coping strategies which both promoted and compromised their T1D management. There was an occasional co-existence of diverse coping strategies (productive and non-productive), and these reflects the personal and contextual stressors they faced. The results call for the need to eliminate barriers of T1D management and equip patients and their caregivers with ongoing T1D coping competencies.

## Background

Type 1 diabetes (T1D) is a complex and demanding chronic health condition. It requires patients to make daily management decisions such as frequent self-monitoring of blood glucose (SMBG), carbohydrate counting, psychological adjustments and insulin injections. Unfortunately, T1D often develops among young people who rarely have the skills and competencies to properly manage this condition [1, 2]. As such, structural stressors during this stage can compound T1D management and affect self-care. Coping and adaptation are inexorable for T1D patients and their caregivers particularly those in resource-limited settings, and therefore critical to our understanding of their health and general well-being.

Lazarus and Folkman [3] defined coping as “constantly changing cognitive and behavioural efforts to manage specific external and/or internal demands that are appraised as taxing or exceeding the resources of the person”. Emanating from their epistemology, existing evidence shows that individual’s approach T1D management either from a problem-focused style (active/productive coping), or an emotional-focused style such as disengagement or avoidance (non-productive coping). Either approach is associated with improved glycaemic control and better quality of life or poor glucose levels and poor quality of life respectively [4–7].

It is known that T1D patients and their caregivers in Ghana face structural barriers including poor access to health care, increased financial burden, limited knowledge, stigma, and discrimination [8, 9]. For instance, the cost of T1D management is staggering, costing between GHC 5–7 a day. Since the daily minimum wage in Ghana is GHC 14.8 (about $1.5USD), the cost of T1D care can drift patients and caregivers into poverty [10]. It is however uncertain how patients and their caregivers cope and adapt to these financial and structural barriers. It is therefore critical to understand how young persons living with T1D and their caregivers cope and adapt to different structural barriers they experience. We sought to provide answers to this question by analysing qualitative evidence on the coping and adaptation strategies adopted by young persons (14–24 years) living with T1D and their caregivers. We theorised our findings within the transactional model of stress and coping.

### Theoretical perspective

The transactional model of stress and coping (TMSC) is a psychological and behavioural theory advanced in 1987 by Lazarus and Folkman [3]. It emphasises stressors and coping as key determinants of human behaviour. The underlying constructs of TMSC are *stress, appraisal, coping efforts, meaning* and *outcomes*. We operationalised *stressors* to denote challenges/barriers which may be individual, social, or structural. Stressors are usually *appraised/evaluated* as either stressful, controllable, or threatening (primary appraisal) [11]. For instance, depending on several T1D patient dynamics including beliefs*,* optimism and family structures, *stressors* may be perceived as controllable or otherwise. Consequently, individuals evaluate stressors and decide whether to cope or not (*secondary appraisal*). This is based on the ability of patients and their caregivers to manage stressors based on factors such as locus of control, self-efficacy, and support groups. For instance, compared to an individual with an external locus of control, an internal locus of control is a buffer against adverse health outcomes [12, 13] and improves adherence to medications [14].

Research evidence on *coping efforts* can broadly be categorised into positive/productive and negative/non-productive efforts. Depending on the stressor, productive coping mechanisms usually include psychological optimism, planning, and social support [15, 16]. Negative coping approaches include persistent non-productive habit formation, spirituality, use of herbal medicine, logistic constraints, behavioural disengagement, and poorer adherence to diabetes regimen [6, 7, 17].

## Methods

### Research and study design

This study was a qualitative inquiry on the coping and adaptation strategies adopted by young people living with T1D and their caregivers in southern Ghana. The study design was descriptive and interpretative phenomenology. Phenomenology entails a description of participants lived experiences and practices based on the meaning they ascribe to the phenomenon (descriptive), to a deeper exploration of underlying reasons and meanings (interpretative). The authors have explored different dimensions of T1D research using phenomenology and has proven to be useful [8]. Scholars inductively explore participants’ meanings of their experiences and organise them into meaningful themes [8].

### Study area and participants selection

The study was conducted in southern Ghana specifically in the Greater Accra, Central and Western regions where T1D is most prevalent [18]. The study area represents a heterogeneous people due to the high migration of people from both the middle and northern belts. The zone also benefits from improved socio-economic infrastructure including major health infrastructure serving as major referral points for most T1D cases in Ghana. Most young people including those from remote areas and their caregivers access healthcare and purchase their T1D management supplies in this zone. The study interviewed 47 participants made up of 28 T1D patients, 12 primary caregivers (PCGs), 6 healthcare providers (HCPs) and Access to Care Manager (ACM) in an insulin distribution company.

The researchers negotiated access to the network of young people living with T1D through the Diabetes Youth Care (DYC) where BAO is affiliated as a Volunteer. PO and BAO introduced the field assistants to the study participants before data collection. This served to facilitate access to young people living with T1D, and to encourage participants to feel comfortable, trust, and share their deep-seated concerns with the research assistants. Participants were identified at DYC meetings, health care facilities and their places of residence and recruited into the study using maximum variation and snowball sampling techniques. The sampling approach enabled participants with different socio-demographic characteristics such as age, sex, and duration of living with T1D to be drawn into the study. Data collection lasted for four weeks in August and September 2021.

Key inclusion criteria entailed a diagnosis of T1D according to the Ghana Health Service guidelines (a range of random venous plasma glucose above 11.1 mmol/L or fasting A1C > 6.0% (6.9 mmol/L) as retrieved from diabetes registers of patients aged between 14 and 24 years and resided in southern Ghana—the inclusion of this diagnostic criteria was for emphasis. PCGs who lived with and HCPs who provided care for a T1D patient aged 14–24 years for not less than 24 months were interviewed. The 24 months continuous were deemed sufficient time to greatly experience the daily life of patients, reduce recall bias, and to provide recent and thick description of events. Data collection and data analysis were continuous to help gauge information power where no new information relating to the subject matter or established criteria on coping emerged.

### Data collection procedure

Ethical clearance was sought from the University of Cape Coast IRB [ID: UCCIRB/CHLS/2021/19]. At the healthcare facility and DYC support group meetings - a support network for young people living with T1D who call themselves *warriors* in Ghana, interviews were granted in small conference rooms. A 2-m interviewing distance was observed between field assistants and participants as a way of observing social distance during the Covid-19 pandemic. In the healthcare setting, informed consent for minors were obtained from caregivers who were present with their child. In some instances, healthcare providers provided proxy consent for minors (aged < 18 years) whose parents/guardians were not present. The assent of minors was sought after seeking parental/guardian informed consent. Interviews were conducted at locations chosen by participants. The study employed four (4) field assistants (FA) to collect data. The FAs had a Master of Philosophy degree (Social Sciences, Public health, and Demography), and had an average duration of two years research experience with qualitative research. The Coordinator of the DYC who has a medical training background was part of the FAs to provide psychosocial support which was found useful. The FAs were trained on qualitative methods, phenomenological study design, studying vulnerable groups, interview guides and data collection for a period of two days, and participated in the data collection.

The study employed unique approaches to study the subject matter amid Covid-19. Data collection methods were both traditional (face-to-face interviews, mystery clients, photovoice) and digital approaches (telephone interviews, video conferencing, asynchronous interviews, and returned emails). These inclusive methodologies were tailored to the unique situations of participants as well as in unison with contemporary research methods during the Covid-19 global pandemic. A maximum of one interview was conducted by each field assistant in a day using English, Fanti or Twi languages which were best spoken and understood by participants. Interviews lasted between 40–120 min and were tape-recorded. The instrument used for the data collection was a semi-structured interview guide. The interviews were transcribed verbatim into the English Language the same day and password protected. Following Wang and Burris [19] methodology, participants were asked to take pictures depicting their lived experiences. The photos drawn from the qualitative project to explore their coping strategies were from three main sources a) three pictures taken by adolescents themselves using their personal smartphones b) a picture taken by a healthcare provider during support group meetings and c) a real-time picture taken by a field officer during interview sessions. All photos adhered to further consenting process from participants and were wilfully shared with the research assistants. The photos selected and presented were those fit for purpose, anonymous, had a voice, represents commonly shared experiences, and agreed by participants for publication. These photos were discussed within four (4) dimensions which are precursors to events, ascribed meaning, lived experiences and aftermath concerns. Participants were compensated for their transportation and airtime costs incurred.

### Data processing, analyses and presentation

The field notes, transcripts and photos were inductively coded independently by the field assistants (BAO and PO) in QSR NVivo 11. The Codebook was shared with DTD, discussed and themes were generated from the codes. Data collection and analyses were done simultaneously. The analytical technique was thematic analysis whereby the authors assigned a word/short phrase that encapsulates codes and photos that were similar in meaning as themes. The steps taken to analyse the data followed Clarke and Braun [20] approaches which were data familiarisation, generating codes, identifying themes, reviewing themes, defining, and naming themes, and locating exemplars. We further included a comparison of different codes/themes via Matrix coding. For instance, comparing participants’ background characteristics with their coping approaches. The findings are presented along themes and supported with photo evidence where appropriate.

### Rigour

Quality assurance mechanisms were embedded in the study design to ensure the collection of valid results during the planning, data collection and analyses phases. *Credibility* measures employed methods such as participants taking of photos that represented their daily lived experiences, prolonged fieldwork, triangulation of data sources, methods and reporting of perspectives, participants re-checking through returned transcripts and independent coding of transcripts. Efforts at ensuring *confirmability* of the results included appropriate research design, as well as methods that have been employed in related studies as suggested by Green, Willis, Hughes et al., [21]. Transcripts were emailed to participants with active email addresses and all healthcare providers to confirm their information. To further ensure that the results were *reliable*, the semi-structured interview guide was informed by a systematic review of the literature and BAO and PO regular interaction with young persons living with T1D and their caregivers. Unlike closed-ended questions, the open-ended questions allowed participants to express their deep-seated concerns about the costs of T1D care and their adaptation strategies. The study made use of acceptable standards and practices of data analysis and reporting that followed the Consolidated criteria for reporting qualitative research (COREQ). We ensured the careful selection of participants using maximum variation sampling techniques. For instance, patients came from either urban or remote areas like their PCGS and therefore represent different socio-economic groups.

## Results

### Background characteristics of study participants

Forty-seven (47) participants representing different target groups were interviewed (See Table 1). Twenty-eight of them were warriors. Within this group, there was equal representation of males and females. Ten of them were aged below 19 years, and they had lived with T1D for about 8 years. Twelve of them reported an immediate family history of diabetes, and most (n=19) of them were students who lived with their primary caregivers (PCGs)—mostly their mothers. All the warriors were actively covered under the NHIS. One of the warriors was married, and two others were cohabiting. Three of the warriors had newly joined the DYC, and seven were irregular meeting attendees. The average age of the PCGs was 45 years, mostly with Junior High School (JHS) and Senior High School (SHS) levels of education. Nine of the 12 caregivers were biological mothers of a warrior, among whom some confirmed they have DM including T1D. Eight of the PCGs were married and mostly engaged in petty trading in nearby markets. PCGs have been engaged in T1D caregiving for about six years. Two out of the six healthcare providers (HCPs) were physicians, nurses, and pharmacists respectively who had been directly engaged in T1D care for the past seven years. An Access to Care Manager (ACM) for an insulin-distributing company participated in the study as well.

**Table 1:** Basic socio-demographic characteristics of participants

**Participant characteristics**	**Participant categories**				**Total (47)**
	**Warriors (28)**	**PCGs (12)**	**HCPs (6)**	**ACM (1)**	
**Sex**					
Female	14	11	3	-	28
Male	14	1	3	1	19
**Age group (in years)**					
14 – 19	10	-	-	-	10
20 – 24	18	-	-	-	18
30 – 39	-	4	3	-	7
40 – 49	-	3	2	1	6
50 and above	-	5	1	-	6
**Duration of living with/providing T1D care**					
Less than 5 years	5	4	1	1	11
5–10 years	17	6	3	-	26
Above 10 years	6	2	2	-	10
**Highest educational level**					
Never Attended	-	1	-	-	1
Primary	2	4	-	-	6
JHS	7	3	-	1	17
SSS/SHS	11	3	-	-	14
Tertiary	8	1	6	-	9
**Family history of DM**					
Yes	12	7	-	-	19
No	13	5	-	-	18
Don’t know	3	-	-	-	3
**Primary caregiver (PCG)**					
None	6	-	-	-	6
Both Parents	3	-	-	-	3
Mother/Grandmother	11	-	-	-	11
Father	3	-	-	-	3
Other relative	3	-	-	-	3
Non-relative	2	-	-	-	2
**PCG Occupation**					
Salary earner	-	2	-	-	2
Petty trader	-	8	-	-	8
Unemployed	-	2	-	-	2
**Religious affiliation**					
Christian	25	11	5	1	42
Muslim	3	1	1	0	5
**Position**					
Physician	-	-	2	-	2
Pharmacist	-	-	2	-	2
Nurse	-	-	2	-	2
ACM	-	-	-	1	1

### Themes

Two major themes which are positive/productive and negative/non-productive coping approaches were identified. Others were the habitual preference to keep T1D a secret and coping with the financial burden of care. Concerning positive coping approaches, three sub-themes which are condition acceptance and planning ahead, social support, and behavioural modifications were identified. Social support included all forms of support received from family, friends and the networks of warriors. The behavioural modifications were largely lifestyle such as diet. Similarly, two sub-themes which are avoidance and disengagement styles, and insulin rationing were identified as negative coping approaches. An adaptational issue that emerged was the habitual preference to keep T1D a secret. Similarly, financial mechanisms/adaptation strategies identified were borrowing money, loans, and the sale of household assets.

### Positive/productive coping approaches

#### Condition acceptance and planning ahead

As indicated, the warriors and their caregivers adopted several positive coping strategies to cope with the plight of T1D management. These positive coping approaches included condition acceptance and planning ahead. We found that the warriors and their caregivers accepted their condition, and always planned their day to avoid “crisis”. In explaining condition acceptance, a mother with 5 years of lived experience had this to say “*I met some who were younger and others older. It was then that I consoled myself that this condition affects the young and old until today”.* Planning ahead took the form of timely renewal of their national health insurance cards, planning about self-monitoring of blood glucose, food arrangements for students in boarding houses, and keeping spare insulin. Concerning planning ahead, the participants made these revelations:

*“I always make sure I have a spare insulin in case the hospital disappoints me”* [a female warrior with 13 years of lived experience].

*All of us in this house have our insurance outdated, but with him, I always ensure that his health insurance is active* [a mother with seven years of lived experience as a caregiver].

A category of young persons who were not able to self-monitor their blood glucose (BG) levels due to a lack of glucometers adopted the habit of periodically checking their BG levels at nearby health facilities at a fee. They then took their BG reading to their homes to inform their insulin dose. In explaining this issue, this was what was said:

*“I don’t have a machine (glucometer). From where I stay to the clinic is not that far, so I go there on a day-to-day except Sundays to check my glucose level at GHC 5 per day”* [a female warrior with seven years of lived experiences].

*“We check, the three of us anytime at a nearby hospital, GHC 5 for each person”* [a mother with six years of lived experience as caregiver of two children living with T1D].

As indicated, caregivers made special food arrangements for students in group quarters, especially those in boarding houses to cater for the nutritional needs of their wards living with T1D. In explaining this issue, this was what was said:

*Daddy knew the headmistress in the school, so he spoke with her and they made arrangement with the kitchen staff to cook for me* [a female warrior with 7 years of lived experience].

*I cooked every week and sent it to her [daughter living with T1D]. The housemistress complained that I am stressing myself and offered to help so I bought the items for her and she prepared food for my daughter* [a mother with 5 years of lived experience of caring for a T1D daughter].

#### Social support

Key sources of social support for the warriors were their parents and HCPs including the DYC. Parents and HCPs provided both relational and instrumental support to the warriors. Instrumental support took the form of buying T1D management logistics, insulin, transportation, and laboratory-related costs. Similarly, expressive support included emotional and psychological support. PCGs also took comfort in the fact they were not the only ones caring for a child living with T1D. Caregivers also received some psychological support from the DYC. PCGs showed optimism in the face of their children living with T1D albeit worries about future uncertainties including untimely death*.* For instance, some PCGS shared that *“I don’t want to show it to him that I am worried and scared, I just act strong and take care of him* [a mother with 7 years of lived experience as a caregiver]*.* In explaining social support, they received, participants had this to say:

*“My mother has been very supportive, sometimes when I want to give up, she encourages me to stay strong”* [a male warrior with 4 years of lived experience].

*I bought the glucometer for her so she can check her sugar levels herself at home to detect whether her sugars are going up or low* [a mother with 5 years of lived experience as a caregiver].

The DYC provided glucometers, test strips and syringes as well as psychosocial support to young persons during monthly support meetings, as well as through online mentorship programmes during the global Covid-19 pandemic. Participants provided insights by saying this, “*Support groups like DYC is one of a kind because they are the other family apart from our relatives* [a male warrior with 7 years of lived experience]”.

Beyond these sources, warriors who were in romantic relationships received support from their dating partners. Whereas others expressed failed relationships due to their T1D, others were happy with the support they received from their boyfriends, who were mostly students of medical professions. In their assertions, this was what was said:

*“My boyfriend knows I am diabetic. He was very protective of me and supportive. Moreover, he was a lab technician, so he was doing all my labs for me when it comes to my sugar* [a female warrior with 5 years of lived experience]*”.*

To buttress this issue, *a* male warrior with 4 years of lived experience had this to say concerning her girlfriend “*She is very supportive, she always tells me not to worry about my diabetes. She gives me money, sometimes GHC 50. Even when I refuse the money, she hides it under my pillow, and it helps with my strips”.*

#### Borrowing and storing insulin

Young persons living with T1D borrowed insulin from their peers living with T1D. Borrowing of insulin was done during periods of insulin shortage at home or healthcare facilities. In their narratives, a male warrior with 4 years of lived experience had this to say:

“*I came back home [due to insulin shortage at the healthcare facility] and went to my friend who is also diabetic and pleaded for a vial of insulin to use. When it got to my turn for monthly review, I took mine [two] and went back and gave him one*”.

**Fig. 1:**
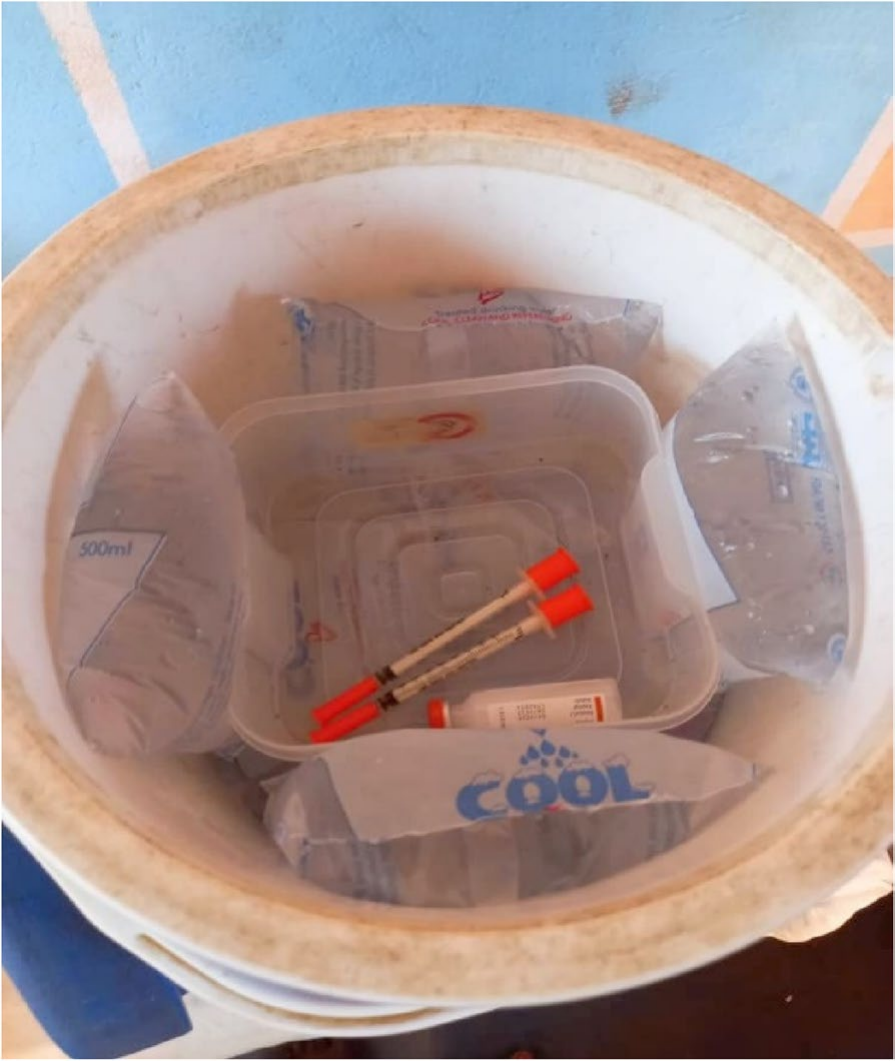
A warrior shows his approach to storing life-saving insulin in a cold flask which he terms as his “refrigerator”. Source: Fieldwork, 16^th^ August 2021

Also, the storage of insulin due to a lack of refrigerators was found to be a major issue, particularly for student warriors in boarding houses (that is students who stay in the school where they are housed and fed by the school authorities). As such, boarding students resorted to the use of flasks/ice-chest with ice blocks to store insulin. Others kept insulin either in a bucket of water, in a teacher’s refrigerator or in the school’s infirmary when refrigerators were available. During photo discussions (Fig. 1), a male warrior with 7 years of lived experience had this to say about this picture he took on his smartphone:

*“I keep it [insulin] in a flask! I make sure*

*I buy ice blocks every day and put it on*

*it to keep it safe”.*

A physician specialist revealed that *“unfortunately, insulin storage is very difficult in places with no fridges, they place the insulin in the coolest part of the room which is usually under the bed or in a ‘cooler’ earthenware pot. Others, if they are lucky, may get a teacher who has a fridge. Many of them have issues in school such that their BG are poorly controlled”.*

### Negative/non-productive/maladaptive coping strategies

Whereas most warriors adopted positive coping strategies to deal with their condition, there was an occasional co-existence of negative coping strategies, particularly by those above age 20 years, and had lived with T1D between 5 and 10 years. As indicated, the three sub-themes identified on negative coping strategies were avoidance and disengagement from insulin therapy, insulin rationing, paying no mind and re-use of syringes.

#### Avoidance and disengagement from continuous insulin therapy

As a response to the daily T1D stress, the pain of injection, abscess, and insulin misconceptions, most warriors at several points in their life disengaged or avoided continuous T1D management. Avoidance and disengagement of insulin, self-monitoring of blood glucose, insulin injection, and reliance on alternative medications were found. In explaining this issue, a female warrior with 7 years of lived experience had this to say:

*Anytime I inject my insulin, the spot gets swollen. I called my father and told him, and he said he was tired of my complaint. So, for over a year, I wasn’t checking my sugar, or injecting my insulin.*

Also, a male warrior with 4 years of lived experience buttressed this issue by revealing that “*I go several weeks without injection, sometimes when I chance upon it [insulin bottle], I just kick it away with my foot.*

#### Insulin rationing

Insulin rationing and failure to monitor BG were commonly found among the warriors. This was their way of managing the shortage or unavailability of life-saving insulin and the cost of test strips. Participants indicated that they reduced their insulin units and skip their BG monitoring due to logistical constraints. In their explanations, a male warrior with 6 years of lived experience made this assertion *“When there is a shortage of insulin, I reduce the unit of insulin shots I take in a day”.* Similarly, a mother with 5 years of lived experience as a caregiver revealed that her child skips her BG monitoring due to the costs of strips.

*“She used to check it [blood glucose] in the morning, afternoon and evening, but now due to the cost of test strips, she checks it in the morning and sometimes in the evening when she feels she ate something she is supposed to avoid”.*

#### Paying no mind and syringes re-use

To avoid the mental strain associated with living with T1D, participants mentally adjusted by paying less attention to the condition. In their assertions, participants had this to say:

*With diabetes, even if you worry, the condition doesn’t go so there is no need to worry about it [a female warrior with 13 years of lived experience].*

*I sometimes must eat foods I am not supposed to eat because that is what we have [a male warrior with 7 years of lived experience].*

As mentioned, re-use of syringes for an average of 3 days were found. Insulin was replaced when injections were painful. For instance, a guardian with 3 years of lived experience as a caregiver revealed that her “daughter” “*changes her syringes after every 4 days”*.

### Keeping T1D a secret – a response to stigma and discrimination

A coping strategy that was identified was the preference to keep T1D a secret. This may be considered “grey” as we acknowledge the complexity in classifying it as either positive/negative coping strategy. To avoid stigma and discrimination, participants adopted a habitual preference of keeping their diagnosis a secret. This was to avoid over-explanation and others being overly-protective, as well as easily get along with others. These assertions confirm this finding:

*I decided to hide mine from the school because I didn’t like the way colleague students and teachers treated my friends who had diabetes* [a male warrior with 10 years of lived experience].

*I didn’t tell him [previous partner] my daughter is sick. For that one, I don’t want anyone to know. Even with the current one [getting ready for re-marriage], he is not aware of my child’s condition. He sees the syringes around and thinks it’s family planning* [a mother with 11 years of lived experience as a caregiver].

Also, T1D patients in boarding schools self-monitored their glucose in hidden places such as washrooms and corners of the school building. For instance, some participants shared this.

*I brought the glucometer out looking for an opportunity to quickly check my sugars, but I was tensed and felt my classmates might see me. So, I put it back and went to the washroom to do my thing [checked BG and took insulin shots]* [a male warrior with 5 years of lived experience].

*He goes there [shop on the school compound] to check* [a mother with 6 years of lived experience as a caregiver for 2 children living with T1D].

However, there were unusual instances where patients willingly confided in others they trusted, or their diagnosis was *mistakenly* known by other people around them. In explaining this issue, a female warrior with 5 years of lived experience had this to say:

*He came to visit me at my place and bought stuffs like Don Simon, Chocolate etc. for me. When he came, my mom and aunty were around so I kept it on a table, and I went out with him to talk. While we were outside my inquisitive aunty opened the package and saw plenty of such stuffs. She shouted and came at him; You want to kill my daughter, don’t you know she is diabetic, then he got to know.*

### Coping with the financial burden of T1D care

Caregivers resorted to borrowing money from neighbours and employers, emotionally appealing to healthcare providers through begging, and loans to cope with the financial burden involved in T1D care. For instance, some participants reported how they borrowed money or took loans from their employers and relatives. In their narrations, this was what was said:

*I go to her [employer] and she gives me money and deduct it from my salary, then I use the money to prepare food for him day by day [a mother with 7 years of lived experience].*

*We sometimes take loans from friends and accept what other family members give us [a male warrior with 7 years of lived experience].*

**Fig. 2:**
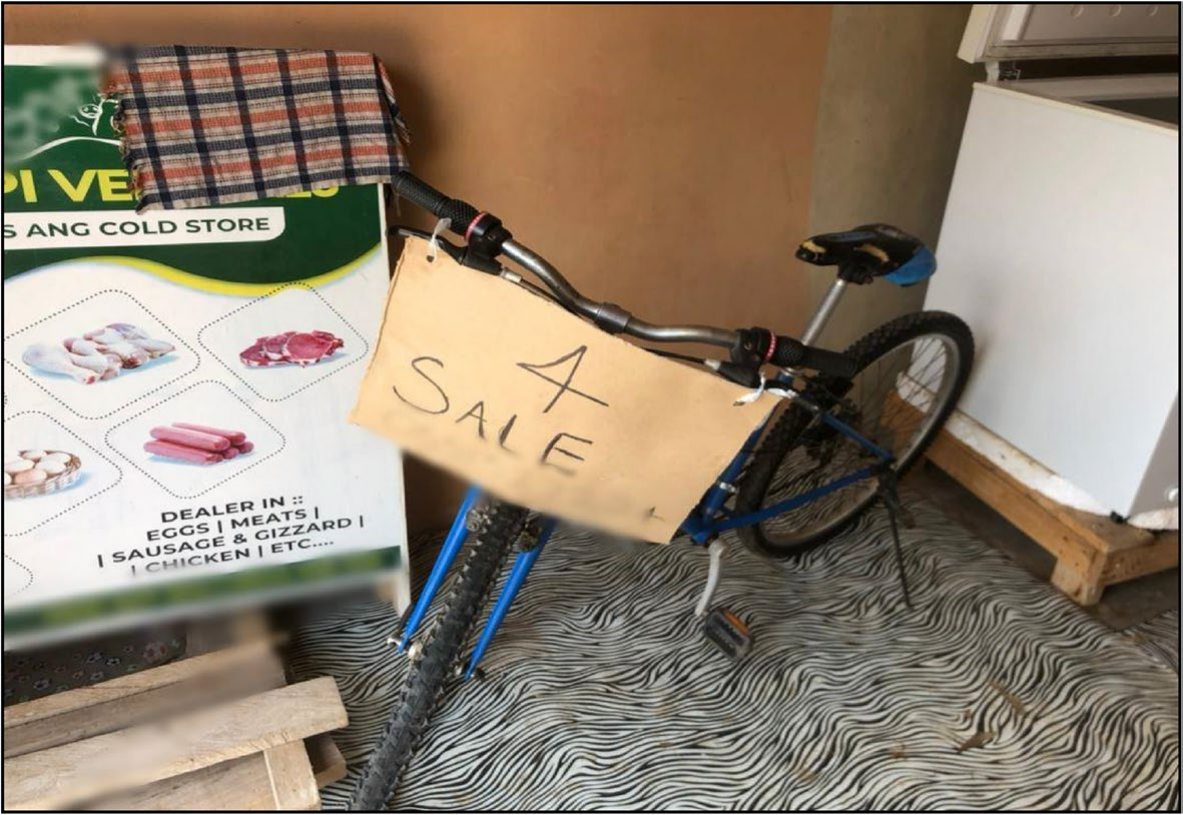
A real-time photo taken by a field assistant to depict the sale of bicycle by a warrior to offset the cost of T1D care. Source: Fieldwork, 30^th^ September 2021

Beyond these adaptation strategies, we found that some PCGs sold household items to offset the cost of care as shown in Fig. 2.

### T1D stress, appraisal, and coping decision making – a conceptual framework

**Fig. 3:**
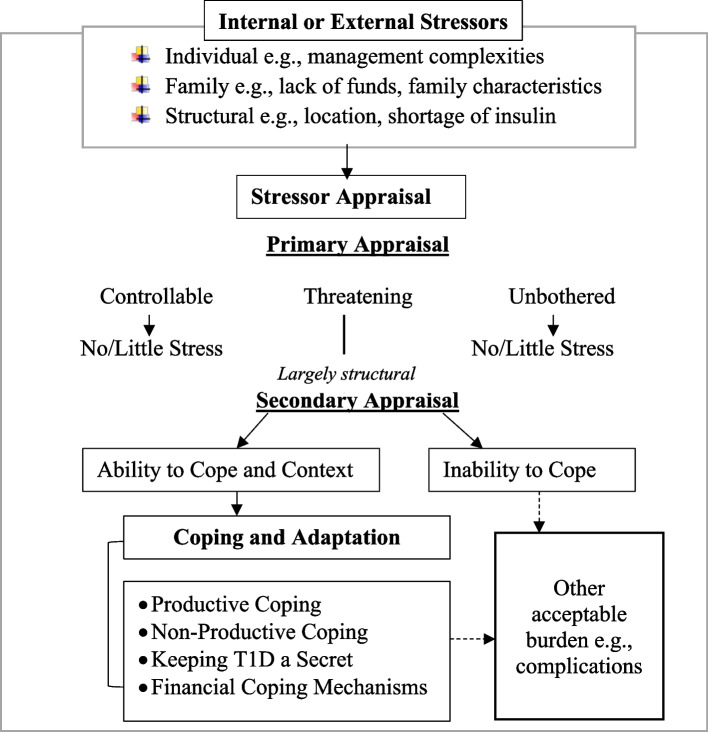
Stressor appraisal and coping decision-making among T1D patients and their caregivers. Source: Authors Construct

In this section, a conceptual framework (Fig. 3) is proposed based on our understanding of T1D stress and coping, and inspired by Lazarus and Folkman’s transactional model on stress and coping theory [3]. The coping strategies identified were influenced by patients and/or caregivers’ appraisal of stressors, which were based on their understanding of T1D in terms of potential consequences of action/inaction, or situations in which they live. In narrative terms, and as shown in Fig. 3, T1D patients and/or their caregivers, especially those in resource-limited areas in Ghana face prevailing stressors/challenges in their efforts to achieve ideal glucose levels. Their ability to cope and/or inability to cope were heavily dependent on their appraisal of stressors as controllable or threatening, and/or favourable or unfavourable environment respectively. Patients and/or their caregivers coping approaches as identified were influenced by the synergy of their coping decision and the context within which coping actions occur. Either of the coping approaches can result in several acceptable burden including unfavourable health outcomes which needs to be explored in further studies.

This conceptual framework has implications for future research and practice. The evidence supports the need to ensure the co-existence of coping competencies and enabling environment for young people living with T1D and their caregivers to adopt productive coping practices. This will include eliminating all forms of barriers such as self and social stigma and discrimination, and improve diabetes health literacy across social systems. T1D competencies will include shaping patients and caregivers’ perception about their T1D health beliefs.

## Discussion

In this study, the coping and adaptation strategies adopted by young persons living with type 1 diabetes (T1D) and their caregivers to deal with the challenges they faced in T1D management were analysed. The study employed qualitative methods including photovoice to unearth participants lived experiences. Photovoice offered an opportunity to explore the lived and subjective experiences of this vulnerable and under-researched population sub-group with imminent health needs and was therefore helpful in unpacking their perspectives. The results show that patients and their caregivers adopted productive, non-productive and other forms of coping strategies which routinely co-existed. Productive coping strategies included planning ahead, seeking social support, and storage of insulin in cold flasks. On the other hand, non-productive coping strategies were avoidance and disengagement, insulin rationing and re-use of syringes. Either of these approaches reflected the multidimensional constraints they faced, and therefore differed from participants in different socio-economic and health circumstances. A plausible productive/non-productive coping strategy was the habitual preference to keep T1D diagnosis and self-care activities such as self-monitoring of blood glucose (SMBG), and insulin injections a secret. As a response to the financial challenges they faced, patients and their caregiver’s borrowed money, insulin, took loans and sold household items.

The findings that warriors and their caregivers adopt productive and maladaptive coping strategies to deal with the challenges of T1D management are consistent with other studies, yet, differed in their novel contribution to coping in resource-limited settings. As a form of productive coping, young people living with T1D manoeuvre through the demanding nature of schoolwork by planning ahead of time [4, 22]. Planning included setting alarms as a self-care strategy [15]. Given that T1D management constraints in resource-limited settings can be pervasive, productive approaches such as condition acceptance and overcoming the barriers of insulin storage can be rewarding. The tenacity of young people living with chronic conditions to constantly evolve to adapt to management constraints can predict better health outcomes. Some studies in advanced countries have found that warriors who planned ahead had higher competence ratings, a better quality of life, and improved metabolic control [4]. In other studies, productive coping such as those found in this study including social support were associated with improved metabolic control [16], and treatment adherence [6].

In home settings, sharing concerns with parents and feeling relief afterwards, talking with other T1D patients/caregivers, as well as relying on the cheers and stories told by friends have proven to be vital [17, 23]. Adolescents living with T1D therefore lean towards partners who can offer them security, support, and assistance in a stable relationship [24]. Social support, self-efficacy, and self-care attitudes enhances self-care behaviour [25] and serves as a psychological pathway towards healthy and sustainable T1D management behaviour. Our findings on psychosocial support corroborates the evidence that social support plays an important role in blood glucose control  [26]. In Ghana, people and formalised organisations in close relations with warriors and their caregivers including DYC were important sources of instrumental and expressive/emotional support. However, unfavourable social environment can have direct and indirect effect (via stress and poor self-care behaviours respectively) on T1D management. Keeping life-saving insulin in a flask as shown in Fig. 1 is a positive coping strategy in T1D resource-limited settings. However, such practices can compromise adequate storage of insulin, and therefore render insulin unsafe/ineffective in regulating blood glucose.

Explicit in the management of T1D was the adoption of non-productive/maladaptive coping strategies including avoidance, disengagement, insulin rationing, and re-use of syringes. Consistent with our findings, young persons living with T1D in Zambia cope by avoiding the stressors [17]. Syringes are designed for single use, yet warriors re-used them for an average of four (4) times, or when injections were found to be painful before it was disposed. This practice is a major risk factor for lipohypertrophy [27]. Unlike insulin, syringes are one of the inexpensive components of SMBG, its re-use could be attitudinal or due to a lack of knowledge, rather than structural concerns which mainly explains insulin rationing. Due to the changing nature of stressors, for example, through demographic, life events and health transitions, coping competencies may be spatio-temporal, and differ from person to person. Again, whereas avoidance and disengagement bothers on psychosocial issues, insulin rationing, and re-use of syringes were responses to the erratic supply of insulin in healthcare facilities, and poor socio-economic status that were found. Maladaptive coping such as those found in this study are known risk factors for poor metabolic and glycaemic control [4, 28]. Several studies have further shown that T1D patients who adopt non-productive coping strategies have higher glycosylated heamoglobin (HbA1c) levels [29, 30].

Contrary to other contexts where patients and their caregivers openly communicated with friends, family, and school authorities about T1D [31], we found that T1D patients and their caregivers kept their condition a secret from their social relations including school authorities largely to avoid stigma and overly-protection as confirmed elsewhere [32, 33]. This result has been confirmed in other studies where parents hesitate to share their future concerns with T1D and were left worried [34]. In terms of recommendations, the habitual preference to keep T1D a secret is a dicey result. Informing others about ones T1D status did not always lead to favourable experiences. This can however be helpful during emergency situations including severe hypoglycaemia. Yet, the manifestation of stigma and discrimination is a barrier to adequate health and well-being, and this justifies their preference to keep their condition and its management a secret. Notwithstanding this finding, we encourage T1D patients to confide among trusted people around them as this may offer long-term benefits.

## Strengths and limitations of the study

The combination of narrative and visual depictions enhances our ability to accurately capture the deep and usually normalised non-productive coping experiences from the participant’s point of view. Readers including policy makers can vicariously reflect on the data themselves. The results particularly the photo-evidence provides some immediate insight into the coping experiences of patients and their caregivers. The study further conceptualises coping decision as it applies to young people living with T1D and their caregivers in resource-limited settings. Despite these strengths, the results from this qualitative study cannot be generalised to other populations not considered in this research.

## Implications for policy, practice, and future research

Healthcare providers should inculcate behavioural coping training into their periodic psycho-social support group meetings for T1D patients, and continuously encourage patients to adopt such competencies into their everyday life, as well as discourage the re-use of syringes by young persons living with T1D. Given that T1D is chronic, there is the need to kick against all forms of individual, structural and systemic stigma and discrimination thereby improving existing social support services for T1D care. The compromises observed in participants coping strategies, including the storage of insulin in unsafe environments calls for the need to identify other social determinants of health that could explain different health outcomes among young people living with T1D with similar access to biomedical care. Inclusion of treatment costs in the government’s health insurance schemes in low- and middle-income countries will contribute to removing the financial barriers to treatment and management of the disease.

## Conclusion

Young persons living with T1D (warriors) and their caregivers adopted coping strategies which both promoted and compromised their T1D management. There was an occasional co-existence of multiple coping strategies—productive and non-productive, to address the personal and contextual stressors they faced. To cope with the financial burden of T1D management, patients, and their caregiver’s borrowed money, sold household items, and took loans from other people. The results calls for the need to equip patients and their caregivers with ongoing T1D coping competencies while eliminate the barriers they faced.

## Data Availability

The transcribed data and/or analysed during the current study is available upon request from the Department of Population and Health, UCC at pop.health@ucc.edu.gh.
